# Abnormal proinflammatory and stressor environmental with increased the regulatory cellular IGF-1/PAPP-A/STC and Wnt-1/β-Catenin canonical pathway in placenta of women with Chronic venous Disease during Pregnancy

**DOI:** 10.7150/ijms.58992

**Published:** 2021-05-27

**Authors:** Miguel A Ortega, Oscar Fraile-Martínez, Miguel A Saez, Miguel A Álvarez-Mon, Ana M Gómez-Lahoz, Coral Bravo, Juan A De León Luis, Felipe Sainz, Santiago Coca, Ángel Asúnsolo, Jorge Monserrat, Luis G Guijarro, Melchor Álvarez-Mon, Julia Bujan, Natalio García-Honduvilla

**Affiliations:** 1 Department of Medicine and Medical Specialities, Faculty of Medicine and Health Sciences, University of Alcalá, 28801 Alcalá de Henares, Spain.; 2 Ramón y Cajal Institute of Sanitary Research (IRYCIS), 28034 Madrid, Spain.; 3 Cancer Registry and Pathology Department, Hospital Universitario Principe de Asturias, 28806 Alcalá de Henares, Spain.; 4 University Center for the Defense of Madrid (CUD-ACD), 28047 Madrid, Spain.; 5 Pathological Anatomy Service, Central University Hospital of Defence-UAH Madrid, 28801 Alcalá de Henares, Madrid, Spain.; 6 Service of Gynecology and Obstetrics, Central University Hospital of Defense-UAH, Madrid, Spain.; 7 Department of Public and Maternal and Child Health, School of Medicine, Complutense University of Madrid, 28040 Madrid, Spain.; 8 Department of Obstetrics and Gynecology, University Hospital Gregorio Marañón, Madrid 28009, Spain.; 9 Health Research Institute Gregorio Marañón, 28009 Madrid, Spain.; 10 Angiology and Vascular Surgery Unit, Central University Hospital of Defense-UAH, Madrid, Spain.; 11 Department of Surgery, Medical and Social Sciences, Faculty of Medicine and Health Sciences, University of Alcalá, 28801 Alcala de Henares, Spain.; 12 Unit of Biochemistry and Molecular Biology (CIBEREHD), Department of System Biology, University of Alcalá, 28801 Alcalá de Henares, Spain.; 13 Immune System Diseases-Rheumatology, Oncology Service an Internal Medicine, University Hospital Príncipe de Asturias, (CIBEREHD), 28806 Alcalá de Henares, Spain.

**Keywords:** Venous insufficiency, Chronic Venous Disease, Pregnancy, IGF-1/PAPP-A/STC-2 pthaway, Wnt/β-catenin canonical pathway

## Abstract

Lower limbs venous insufficiency refers to a wide variety of venous disorders grouped by the term of chronic venous disease (CVD). Hemodynamic and hormonal changes related to pregnancy period, may promote the development of CVD affecting approximately 1 in 3 women. It has been shown that the presence of this condition is associated with damage and placental suffering. Thus, taking IGF-1/PAPP-A/STC-2, inflammatory cytokines production, PI3K/Akt and Wnt/ β-catenin pathways as a part of the alterations that occurs in the placenta due to CVD, the aim of this study will be to examine the main components of these pathways. Genic and protein expression of PAPP-A, STC-2, IGF-1, IRS-4 Wnt-1, β-catenin, c-myc, Cyclin D1, IL-4/IL-6 and PI3K/Akt/mTOR pathway will be analysed through RT-qPCR and immunohistochemical techniques in women with CVD (n=62) and pregnant women without this condition (HC) (n=52). PAPP-A, IGF-1, IL-4, IL-6, IRS-4, PI3K, Akt, mTOR, Wnt-1, β-catenin, c-myc and Cyclin D1 expression were found to be increased in women with CVD, whereas STC-2 were decreased in this group, compared to non-affected women. Our study has demonstrated that IGF-1/PAPP-A/STC-2 axis, PI3K/Akt and Wnt/β-catenin pathways, along with c-myc, Cyclin D1 and inflammatory cytokines are altered in placenta women with CVD. These results extent the knowledge that CVD is associated to a placenta damage with abnormal tissue environment and cellular regulation.

## 1. Introduction

Lower limbs venous insufficiency refers to a plethora of venous disorders, grouped under the term of Chronic Venous Disease (CVD), being the varicose vein its most important clinical manifestations 1. It is a condition with a high prevalence in western societies, where approximately 60-70% of adults may present CVD 2, 3. Annual incidence varies from 2.6% in women to 1.9% in men, according to the Framingham study 4. Numerous risk factors could promote the onset of CVD, such as female gender, genetic predisposition, smoking, obesity, or pregnancy 5, 6. Women´s body undergoes a wide range of changes during pregnancy which are crucial for the fetal development, mainly due to hormonal and hemodynamic alterations, importantly affecting the vascular system 7-9. It has been described that up to a 28% of women could present CVD during gestation, generally in the third trimester 7, 10-12.

Recent studies have demonstrated how pregnancy related CVD is associated with the presence of placental suffering and damage markers, finding an increase in processes of angiogenesis and lymphangiogenesis, hypoxia and oxidative stress 12-15. Worthy of note is the fact that gestational CVD has been linked with higher levels of systemic lipid peroxidation in these women, along with a fetal pH acidification in the newborns 15. Equally, these alterations have also been described in severe vascular pathologies such as preeclampsia 16-18 or fetal growth restriction 19, thus showing the importance of continue deepening in the pathophysiological mechanisms of placental damage in women with CVD.

In this sense, it is known that placenta is an essential organ for the maternofoetal exchange of nutrients and waste products, with important functions in the metabolism and in the release of many substances 20, 21. In this line, the expression of pregnancy-associated plasmatic protein A (PAPP-A) has been shown to be of great relevance in the placental tissue, where it is synthesised, depending its serum levels of a broad variety of factors such as age, ethnic, weight and physiological or pathological status of the mother 22. PAPP-A, also known as pappalysin 1 is a metalloprotease belonging to the superfamily of metzincins, and its activity plays a key role during pregnancy, increasing the bioavailability of insulin-like growth factors (IGF) 1 and 2 23. PAPP-A degrades the IGF binding proteins (IGFBP) 4 and 5, being IGFBP-4 its main target. Consequently, raise free IGF levels within the tissue, binding to the IGF-1 receptor (IGF-1R), hence leading to a signalling cascade mediated by IGF 24. It has been shown how PAPP-A overactivation may induce an inflammatory response, mostly conducting to a cytokine production and release through the PI3K/Akt pathways, regulated by IGF signalling 25. In addition, binding of IGF to its receptor, triggers the activation of downstream products like insulin receptor substrate 4 (IRS-4), Wnt/β-catenin canonical pathway, c-myc or Cyclin D1, with mitogenic effects 26-28. On the other hand, stanniocalcin-2 (STC-2) is an important component of IGF axis, acting as a negative regulator of the proteolytic activity of PAPP-A in the tissue 29. Proper homeostasis of trophic factors such as IGFs are crucial for the development and functions of the placenta, and their dysregulation have been shown to be related with the pathogenesis of some pregnancy complications 30.

Many authors have revealed the alterations of PAPP-A expression levels and all its cascade in vascular pathologies during pregnancy like preeclampsia, with serious implications in the maternofoetal health 31, 32. Recently, Wang et al. 33 have showed how genic and protein expression of Wnt/β-catenin components is altered in the placental tissue of women with this condition. Likewise, PI3K/Akt pathway have also been observed to play an important role of the pathogenesis of preeclampsia 34. For that reason, it is necessary to evaluate the status of PAPP-A and their subsequent cell signalling through the analysis of the genic and protein expression in the placenta of women presenting gestational CVD.

## 2. Patients and Methods

### 2.1. Study population

An observational, analytical and prospective cohort study was accomplished, comprising 114 women in the third trimester of pregnancy (32 weeks). 62 were clinically diagnosed with CVD, being median age 33[22 to 40] years old, and gestational period 40.5 [39 to 41.5] weeks. Other 52 patients were studied in parallel as controls, being median age 34[27 to 41] years old, and gestational period 41[39 to 42] weeks. This study has been performed according to basic ethical principles: autonomy, beneficence, nonmaleficence and distributive justice. Its development pursued Good Clinical Practice guidelines, principles announced in Declaration of Helsinki (2013) and Oviedo Convention (1997). Patients were informed and each one of them signed the pertinent informed consent. The Project was approved by Clinical Research Ethics Committee from Central Hospital of Defense Gómez Ulla - UAH (37/17) in March of 2017. During consultation in third trimester it is conducted a medical history, general physical examination and exploration of the lower limbs by Eco-Doppler (Portable M-Turbo Eco-Doppler; SonoSite, Inc., Washington, EEUU) at 7,5 MHz.

Inclusion criteria were women older than 18 years old in their third trimester of pregnancy with clinical evidence of CVD in lower limbs, with a CEAP (Clinical-Etiology-Anatomy-Pathophysiology) classification of 1 or higher 35. Exclusion criteria were women with Diabetes mellitus and Endocrine disorders, Arterial Hypertension (AHT), autoimmune diseases, active infectious diseases, venous malformations, cardiac insufficiency, renal or pulmonary, preeclampsia and/or Hellp syndrome, intrauterine growth restriction due to unknown causes, body mass index (BMI) ≥ 25kg/m^2^, toxic habits, pathological lesions such as placental infarction, avascular villi, delayed villous maturation, chronic villitis, and appearance of any other of the previous exclusion criteria during the following months, previous evidence of VI.

### 2.2. Samples from placental tissue

Placental tissue biopsies were obtained once placenta was expelled. In all cases, 5 fragments were obtained from placenta using a scalpel to ensure that samples included multiple cotyledons. These fragments were introduced in two different sterile tubes: one containing Minimum Essential Medium MEM) with 1% antibiotic/antimycotic (both from ThermoFisherScientific, Waltham, MA, EEUU) and the other one containing RNAlater® solution (Ambion, Austin, TX, EEUU). In the laboratory, the samples were processed in a laminar flow bench II Telstar AV 30/70 Müller 220 V 50 M Hz (Telstar SA Group, Terrassa, Spain), in a sterile environment. The preserved samples were maintained in 1 mL RNAlater®, -80ºC until gene expression analysis were accomplished. Samples preserved in MEM were destined to histological and immunodetection studies. Samples preserved in MEM were washed and hydrated multiple times with médium without antibiotic to eliminate blood cells and were cut in fragments that were fixed in F13 (60% ethanol, 20% methanol, 7% polyethylene glycol, 13% distilled H2O), following established protocols (15). Once included, paraffin embedded tissue blocks are prepared with molds. When paraffin is solidified, with a rotary microtome HM 350 S (Thermo Fisher Scientific, Massachusetts, EEUU) several 5 μm sections are obtained, and these are stretched in a hot water bath and are collected in a glass microscope slide, previously treated with polylysine 10% for a better coating.

### 2.3. Gene expression studies using RT-qPCR

RNA was extracted using acid guanidinium thiocyanate-phenol-chloroform extraction method described by Ortega et al 36. RT-qPCR was performed in StepOnePlus™ System (Applied Biosystems - Life Technologies, Waltham, Massachusetts, EEUU), using standard curve method. Reaction was accomplished this manner: dilution 1:20 of 5 μl from each simple in free nucleases water and mixed with water without DNase and RNase in MicroAmp® 96-well plate (AppliedBiosystems - Life Technologies) for a reaction with a total volume of 20 μl. All sequences were designed *de novo* (Table 1), using Primer-BLAST 37 and AutoDimer 38.

### 2.4. Protein expression studies by immunohistochemistry

Antigen-antibody reaction was detected by avidin biotin complex method (ABC) with peroxidase as chromogen, according to Ortega et al. 39 protocol´s. Incubation with primary antigen was diluted in BSA 3% and PBS during the whole night at 4ºC (Table 2.A). Incubation with secondary antigen linked to biotin and diluted in PBS was performed during an hour and a half at ambient temperature (Table 2.B). The peroxidase-avidin conjugate ExtrAvidin®-Peroxidase (Sigma-Aldrich, St. Louis, MO, EEUU) was used during 60 minutes at ambient temperature (1:200 dilution in PBS), revealing with diaminobenzidine chromogenic substrate (Kit DAB, SK-4100, Vector Laboratories, Burlingame, CA, EEUU). Chromogenic substrate was prepared immediately before exposure (5mL distilled H2O, 2 drops of buffer, 4 drops of DAB, 2 drops of hydrogen peroxide). This technique allows a brown dyeing. In all immunochemistry studies, sections from the same tissue were used as negative control, whose incubation with primary antibody was substituted for incubation with blocking solution (PBS).

### 2.5. Statistical analysis and results interpretation

For statistical analysis, software GraphPadPrism® 6.0 was used. Mann-Whitney U statistical test was applied. Data are expressed as median with interquartile range (IQR). Significance p-value defined was p<0,05 (*), p <0,01 (**), p <0,001 (***). For every patient in every established group, 5 sections and 10 fields per section were examined by random choice.

Patients were described as positives when marked mean area in the sample was greater or equal to 5% from total, following anatomopathological protocol by Cristóbal et al. 40. Slides were examined under Zeiss Axiophot (Carl Zeiss, Germany) optical microscope.

## 3. Results

### 3.1. Patients with gestational CVD show an increase in PAPP-A and IGF-1 expression in placental villi

**Figure 1:**
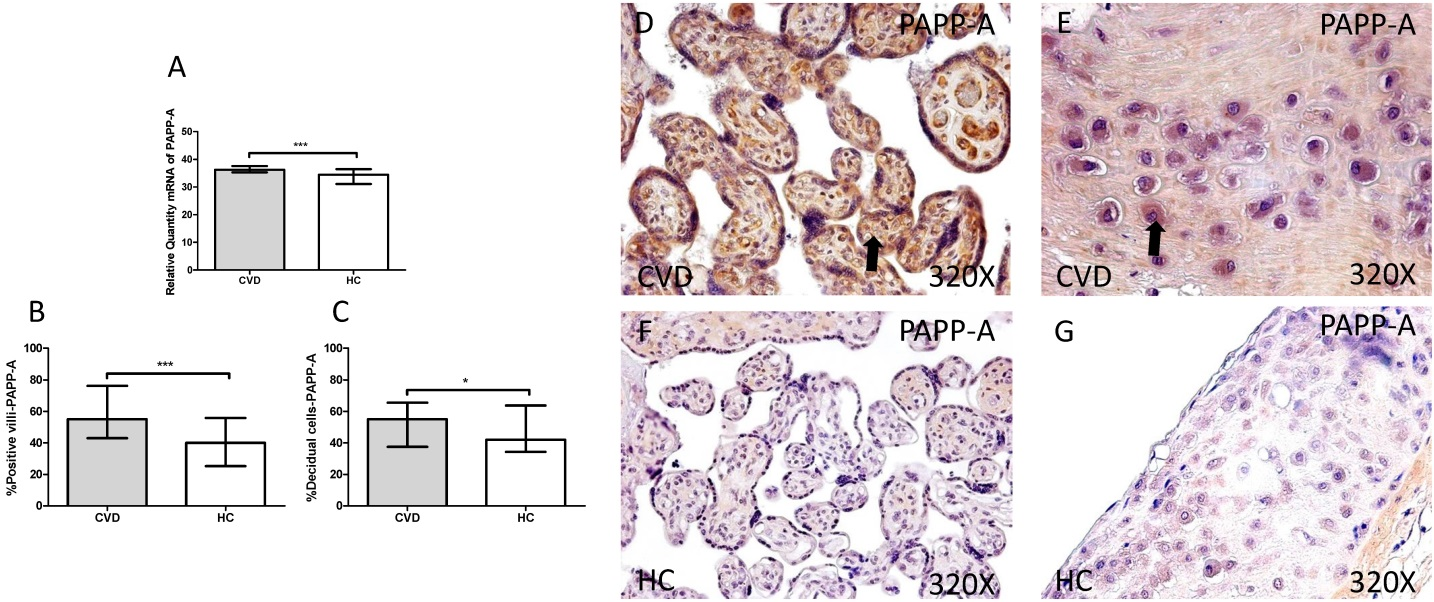
** A.** RNA expression levels for PAPP-A by RT-qPCR. **B.** Percentage of placental villi with positive protein expression for PAPP-A by using immunochemistry techniques. **D-G.** Images where immunoexpression of PAPP-A is showed in placental villi (D and F) and in decidual cells (E and G). CV D=Women with diagnosed gestational chronic venous disease. HC= Venous control p<0.05 (*), p<0.01 (**), and p<0.001 (***). Arrow=shows positive expression of the protein.

First we analyzed the IGF-1/PAPP-A/SCT-2 pathway expression in placentas of women gestational CVD and in controls HC by using RT-qPCR and immunochemistry. Our results showed a significant increase in PAPP-A gene expression in placentas of patients with CVD, expressed in arbitrary units (CVD=36.213 [18.563-40.890], HC=32.438 [15.203-39.895], ***p=0.003, Figure 1.A). Protein expression analysis showed a significant increase in placental villi percentage positive for PAPP-A in patients with CVD (CVD=55.000 [12.000-99.000], HC=40.000[10.000-86.000] ***p=0.0003, Figures 1.B and D-F). Besides, decidual cells percentage with positive expression for such protein was significantly higher in placentas of women with gestational CVD [CVD=55.000 [20.000-88.000], HC=42.000[19.000-84.000], *p=0.0297, Figures 1.C and E-G].

**Figure 2:**
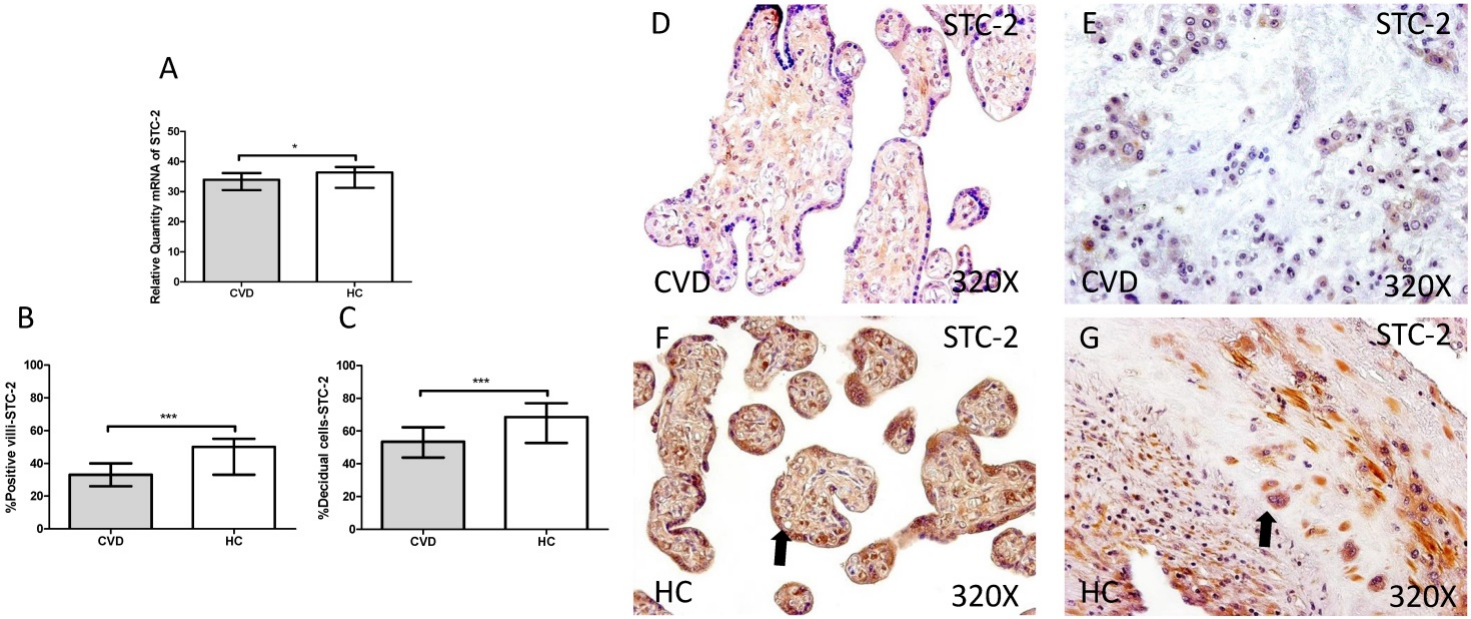
** A.** mRNA expression levels for STC-2 by RT-qPCR. **B.** Percentage of placental villi with positive protein expression for STC-2 by using immunochemistry techniques.** D-G.** Images where immunoexpression of STC-2 is showed in placental villi (D and F) and in decidual cells (E and G). CVD=Women with diagnosed gestational chronic venous disease. HC= Venous control p<0.05 (*), p<0.01 (**), and p<0.001 (***). Arrow=shows positive expression of the protein.

Our study showed a significant decrease in SCT-2 gene expression in placenta of women gestational CVD in comparison to HC (CVD=33.992 [19.068-49.124], HC=36.389 [20.560-40.256], *p=0.0376, Figure 2.A). Immunochemistry study showed a decrease in the percentage of positive villi for SCT-2 in placentas of women with gestational CVD (CVD=33.000 [13.000-55.000], HC=50.000 [20.000-85.000], ***p<0.0001, Figure 2.B and D-F), just like decidual cells percentage (CVD=53.500 [12.000-91.000], HC=68.500 [22.000-98.000], ***p=0.0002, Figures 2.C and E-G).

**Figure 3:**
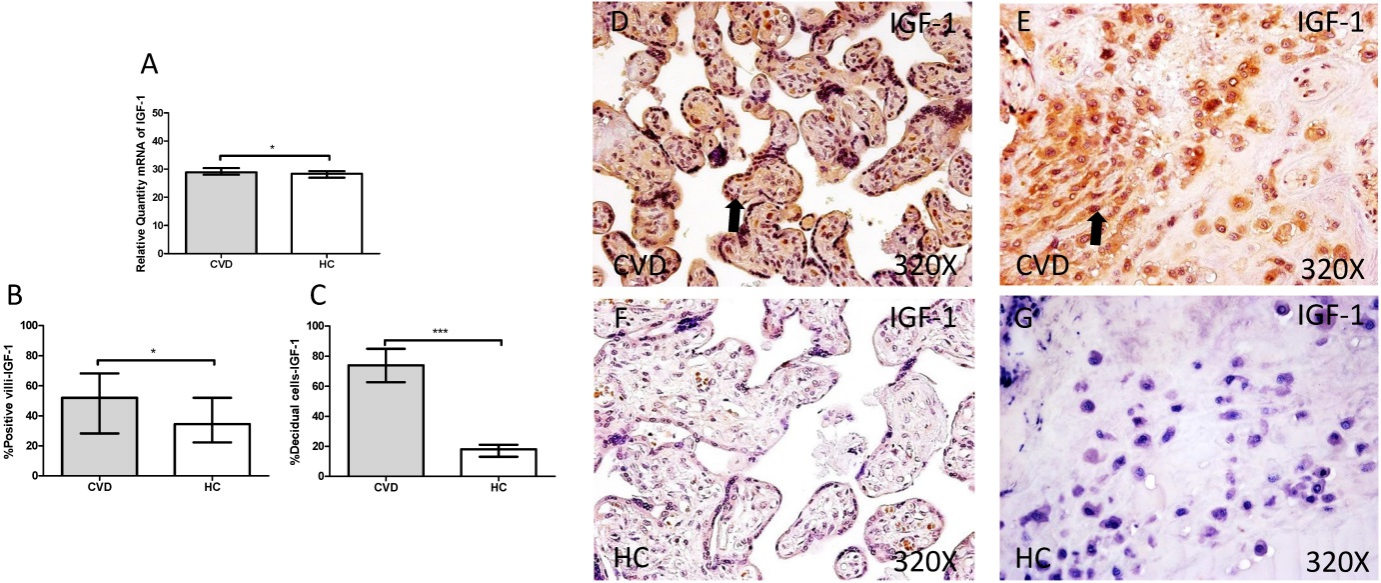
** A.** mRNA expression levels for IGF-1 by RT-qPCR. **B.** Percentage of placental villi with positive protein expression for IGF-1 by using immunochemistry techniques. **D-G.** Images where immunoexpression of IGF-1 is showed in placental villi (D and F) and in decidual cells (E and G). CVD=Women with diagnosed gestational chronic venous disease. HC= Venous control p<0.05 (*), p<0.01 (**), and p<0.001 (***). Arrow=shows positive expression of the protein.

The study of IGF-1gene expression showed a significant increase in placenta of women gestational CVD in comparison to HC (CVD=30.902 [19.068-49.134], HC=28.394 [7.759-39.889], *p=0.0188, Figure 3.A). In parallel, a significant increase was observed in IGF-1 protein expression in placental villi of women with gestational CVD (CVD=52.000 [13.000-99.000], HC=34.500 [9.000-97.000], *p=0.0210, Figure 3.B and D-F), as well as in decidual cells of placentas of women with gestational CVD (CVD =74.000 [13.000-99.000], HC=18.000 [4.000-46.000], ***p<0.0001, Figure 3.C and E-G).

### 3.2. Increase in IRS-4 expression in placentas of women with gestational CVD

**Figure 4:**
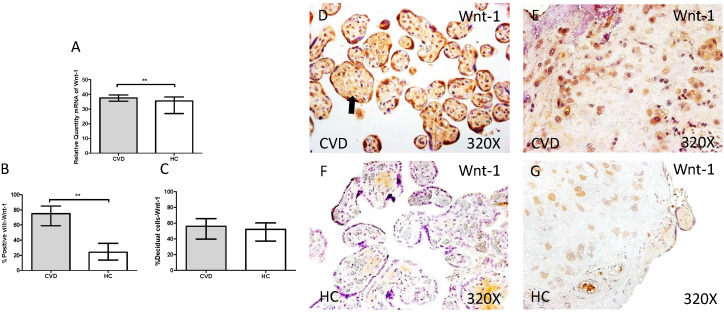
** A.** Expression levels of mRNA for IRS-4 by RT-qPCR. **B.** Percentage of placental villi with positive protein expression for IRS-4 by using immunochemistry techniques. **D-G.** Images where immunoexpression of IRS-4 is showed in placental villi (D and F) and in decidual cells (E and G). CVD=Women with diagnosed gestational chronic venous disease. HC= Venous control p<0.05 (*), p<0.01 (**), and p<0.001 (***). Arrow=shows positive expression of the protein.

Next, we investigated cell transduction signal related to IGF-1 and PAPP-A stimulation. We observed a significant increase in IRS-4 gene expression in placentas of women with gestational CVD in comparison to HC (CVD =34.986 [18.826-45.852], HC=30.386 [11.969-44.028], *p=0.0120, Figure 4.A). Immunochemistry studies showed how placental villi percentage with positive expression for IRS-4 was significantly enhanced in patients with gestational CVD (CVD =75.500 [32.000-99.000], HC=36.000 [14.000-81.000], ***p<0.0001, Figure 4.B and D-F), just like percentage of decidual cells for these patients (CVD =74.500 [34.000-99.000], HC=34.000 [14.000-72.000], ***p<0.0001, Figure 4.C and E-G).

### 3.3. Placenta of women with gestational CVD show an activation of PI3K/Akt/mTOR pathway

**Figure 5:**
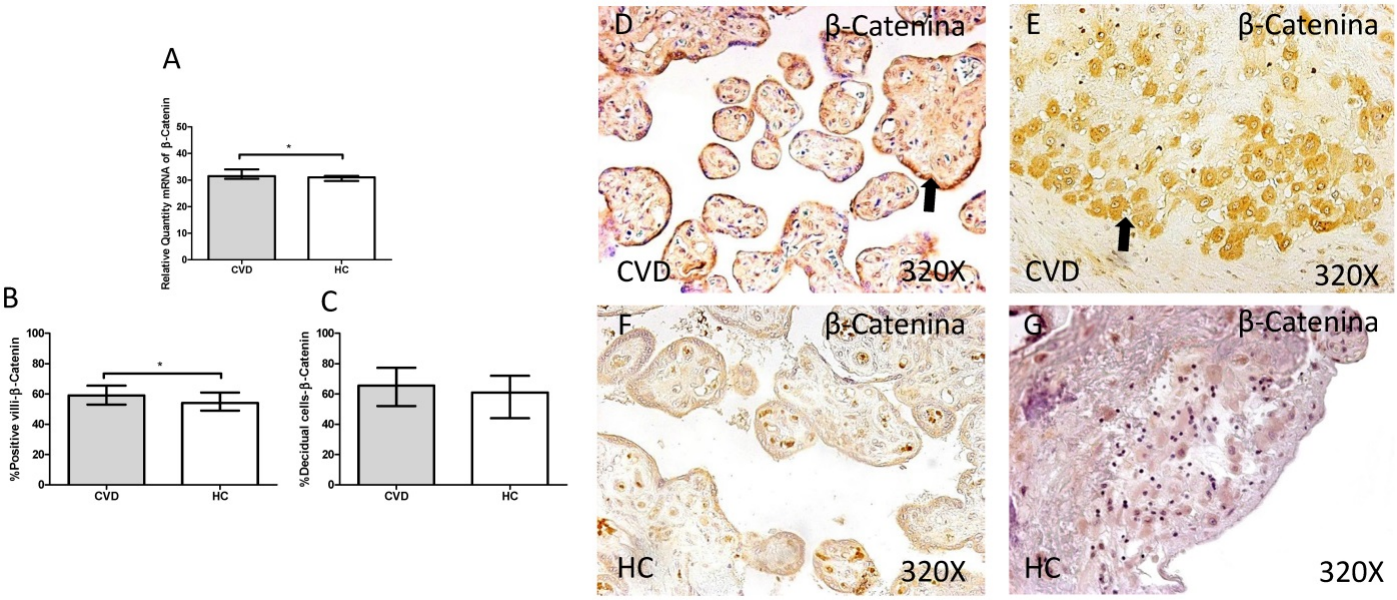
** A.** mRNA expression levels for PI3K by RT-qPCR. **B.** Percentage of placental villi with positive protein expression for PI3K by using immunochemistry techniques. **D-G.** Images where immunoexpression of PI3K is showed in placental villi (D and F) and in decidual cells (E and G). CVD=Women with diagnosed gestational chronic venous disease. HC= Venous control p<0.05 (*). Arrow=shows positive expression of the protein.

**Figure 6:**
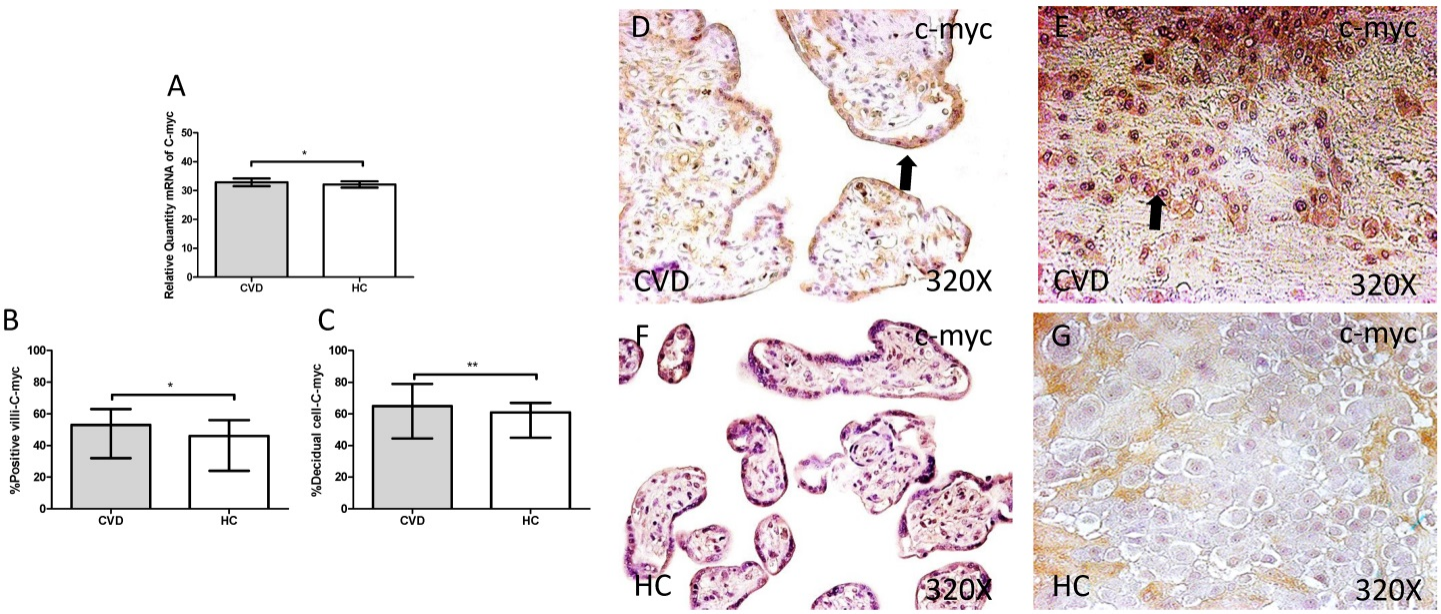
** A.** mRNA expression levels for Akt by RT-qPCR. **B.** Percentage of placental villi with positive protein expression for Akt by using immunochemistry techniques. **D-G.** Images where immunoexpression of Akt is showed in placental villi (D and F) and in decidual cells (E and G). CVD=Women with diagnosed gestational chronic venous disease. HC= Venous control p<0.05 (*), p<0.01 (**), and p<0.001 (***). Arrow=shows positive expression of the protein.

**Figure 7:**
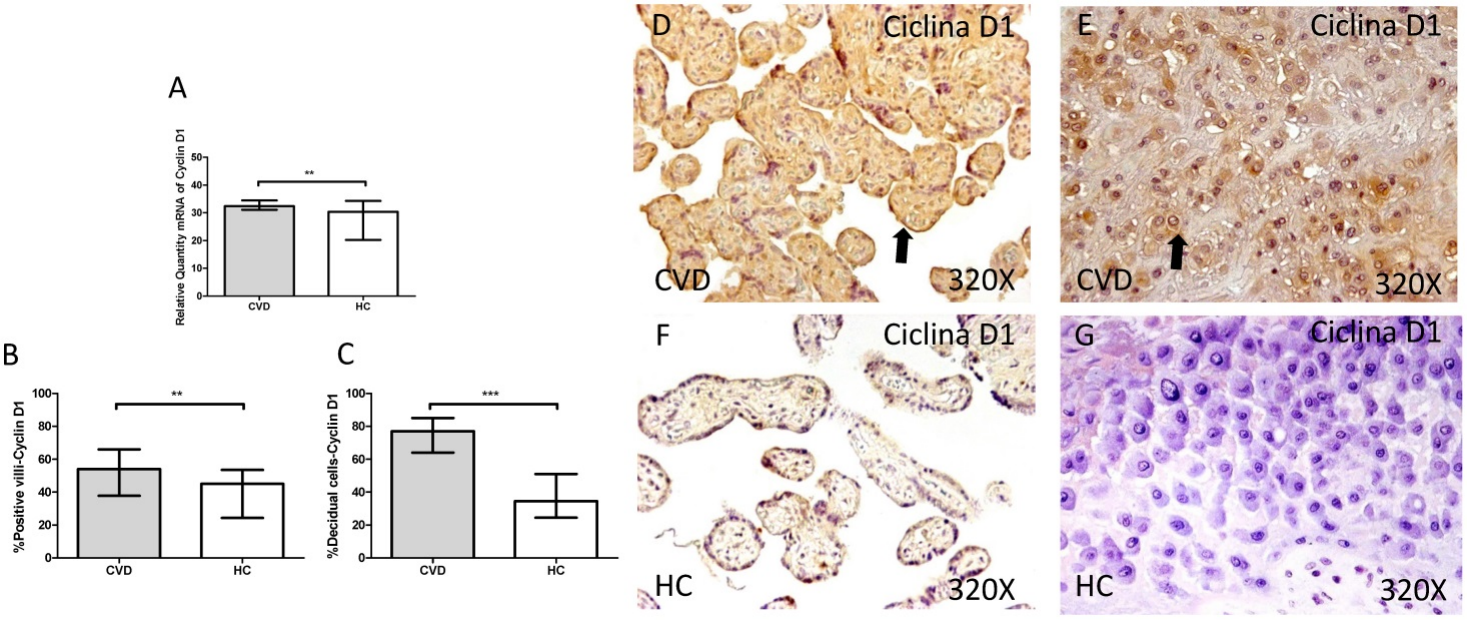
** A.** mRNA expression levels for mTOR by RT-qPCR. **B.** Percentage of placental villi with positive protein expression for mTOR by using immunochemistry techniques. **D-G.** Images where immunoexpression of mTOR is showed in placental villi (D and F) and in decidual cells (E and G). CVD=Women with diagnosed gestational chronic venous disease. HC= Venous control p<0.05 (*), p<0.01 (**), and p<0.001 (***). Arrow=shows positive expression of the protein.

The gene expression study showed a significant increase in PI3K / Akt / mTOR in placentas of women with gestational CVD [(PI3K=CVD=27.729[12.966-43.915], HC=24.536[10.190-43.915], *p=0.0236, Figure 5.A); (Akt=CVD=34.063[17.542-46.616], HC=24.723 [10.566-44.562], **p=0.0015, Figure 6.A); (mTOR=CVD=31.925 [13.033-47.592], HC=25.079[10.490-43.069], **p=0.0040, Figure 7.A)].

Immunohistochemical studies show a significant increase in placental villi with positive protein expression for PI3K in the placentas of women with CVD (CVD = 55,000 [15,000-94,000], HC = 48,500 [16,000-87,000], * p = 0.0333, Figure 5.C and D). PI3K expression in decidual cells was also significantly increased (CVD = 58,000 [26,000-87,000], HC = 52,000 [21,000-86,000], p = 0.0378, Figure 5.C).

Furthermore, Akt protein expression showed a significant increase in the placental villi of patients with gestational CVD (CVD = 64,500 [21,000-97,000], HC = 51,500 [14,000-87,000], ** p = 0.0035, Figure 6.B and D). Similarly, Akt protein expression in decidual cells was significantly higher compared to HC (CVD = 61,000 [31,000-89,000], HC = 47,000 [13,000-81,000], ** p = 0.0023, Figure 6. C and E).

Furthermore, mTOR showed an increase in protein expression in placental villi with CVD (CVD = 62,500 [18,000-97,000], HC = 45,000 [12,000-94,000], ** p = 0.0016, Figure 7.B and D). It was similarly observed in the decidual cells of CVD placentas (CVD = 63,000 [21,000-98,000], HC = 45,500 [14,000-86,000], ** p = 0.0015, Figure 7.C and E).

### 3.4. Placentas of women with gestational CVD show an increase in Wnt-1/β-catenin pathway

**Figure 8:**
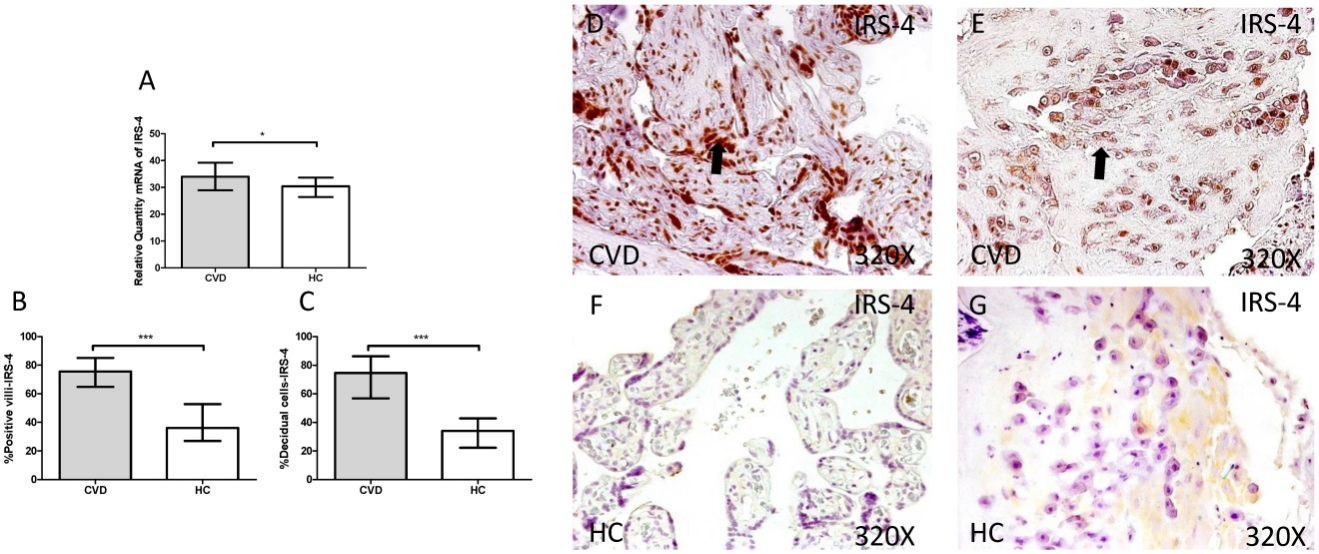
** A.** mRNA expression levels for Wnt-1 by RT-qPCR. **B.** Percentage of placental villi with positive protein expression for Wnt-1 by using immunochemistry techniques. **D-G.** Images where immunoexpression of Wnt-1 is showed in placental villi (D and F) and in decidual cells (E and G). CVD=Women with diagnosed gestational chronic venous disease. HC= Venous control p<0.05 (*), p<0.01 (**), and p<0.001 (***). Arrow=shows positive expression of the protein.

In parallel, we also studied the Wnt-1/β-catenin canonic pathway in placenta of women with gestational CVD and HC. The genic expression analysis for Wnt-1 showed a significant increase in placentas of women with gestational CVD ain comparison to HC patients (CVD=37.549 [21.987-49.585], HC=35.540 [5.865-39.678], **p=0.0026, Figure 8.A). In this sense, placental villi percentage positive for Wnt-1 expression was observed as significantly raised (CVD=75.000 [33.000-98.000], HC=24.000 [6.000-63.000], ***p<0.0001, Figure 8.B and D-F). There were not significant differences observed in percentages of Wnt-1 expression in decidual cells from the study groups (CVD=56.000 [21.000-97.000], HC=52.000 [10.000-96.000], p=0.1762, Figure 8.C and E-G).

**Figure 9:**
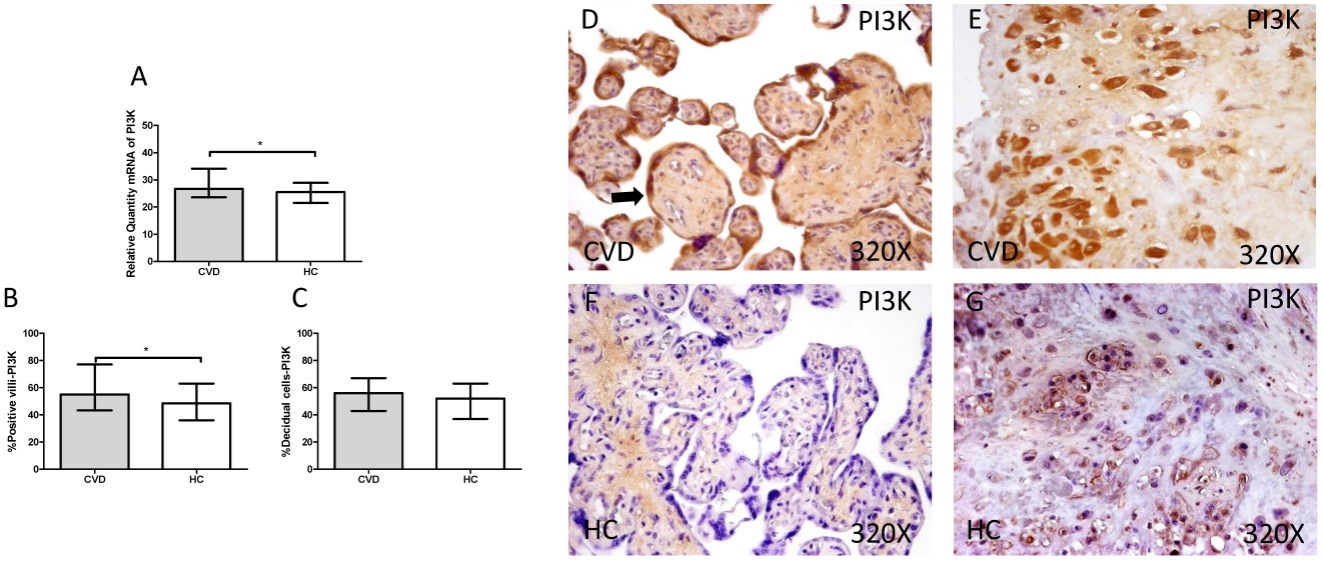
** A.** mRNA expression levels for β-catenin by RT-qPCR. **B.** Percentage of placental villi with positive protein expression for β-catenin by using immunochemistry techniques. **D-G.** Images where immunoexpression of β-catenin is showed in placental villi (D and F) and in decidual cells (E and G). CVD=Women with diagnosed gestational chronic venous disease. HC= Venous control p<0.05 (*), p<0.01 (**), and p<0.001 (***). Arrow=shows positive expression of the protein.

In addition, a significant increase in gene expression of β-catenin was observed in placentas of women with gestational CVD in comparison to HC (CVD=31.464 [28.811-45.655], HC=30.967 [20.572-39.710], *p=0.0143, Figure 9.A). Protein expression analysis showed how the percentage of placental villi with positive expression for β-catenin was significantly higher in placentas of women with gestational CVD (CVD=59.000 [25.000-81.000], H=54.000 [21.000-79.000], *p=0.0288, Figure 9.B and D-F). There were no significant differences observed in percentage of decidual cells with positive expression for β-catenin among the established study groups (CVD=65.500 [22.000-99.000], HC=61.000 [15.000-85.000], p=0.0900, Figure 9.C and E-G).

**Figure 10:**
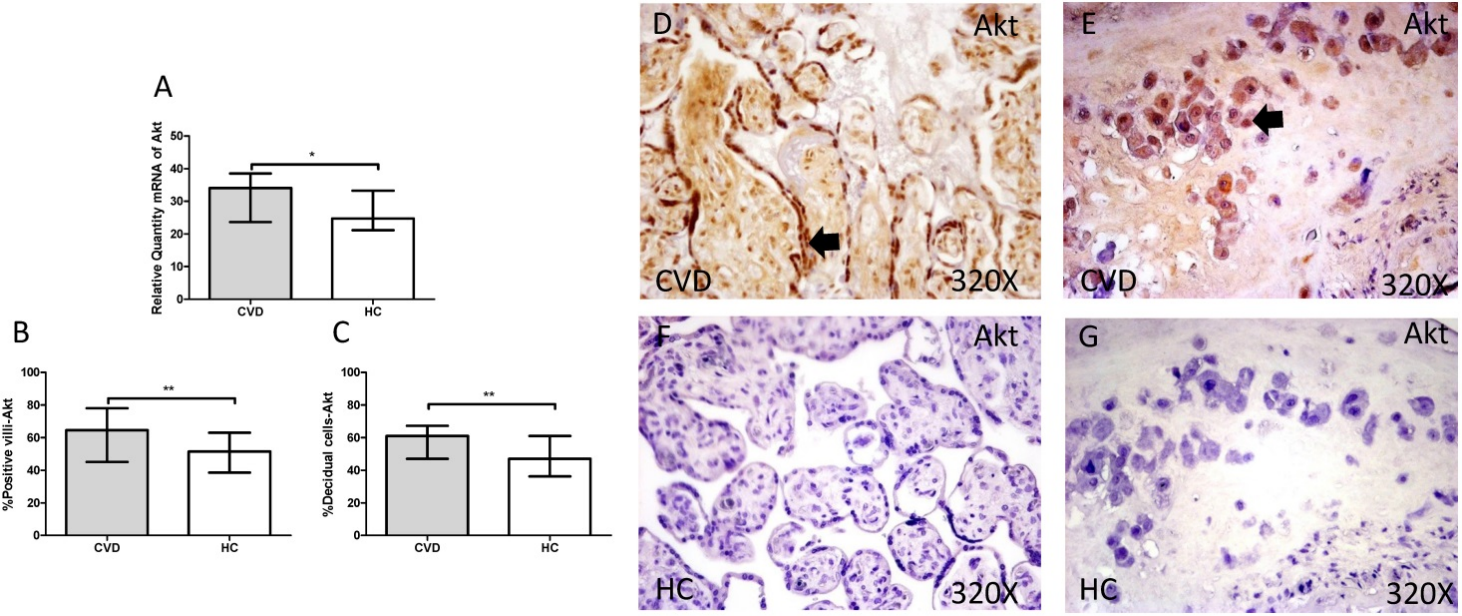
** A.** mRNA expression levels for c-myc by RT-qPCR. **B.** Percentage of placental villi with positive protein expression for c-myc by using immunochemistry techniques. **D-G.** Images where immunoexpression of c-myc is showed in placental villi (D and F) and in decidual cells (E and G). CVD=Women with diagnosed gestational chronic venous disease. HC= Venous control p<0.05 (*), p<0.01 (**), and p<0.001 (***). Arrow=shows positive expression of the protein.

The study of c-myc genic expression by RT-qPCR showed a significant increase in placentas of women with gestational CVD comparing to HC (CVD=32.836 [21.233-49.982], HC=32.103 [20.710-37.950], *p=0.0360, Figure 10.A). There were a significant differences observed in the percentage of placental villi (CVD=53.000 [9.000-96.000], HC=46.000 [7.000-82.000], *p=0.0463, Figure 10.B and D-F). and decidual cells positive for c-myc expression in placentas of women with gestational CVD in comparison to the HC group (CVD=65.000 [34.000-99.000], HC=61.000 [21.000-85.000], *p=0.0284, Figure 10.C and E-G).

**Figure 11:**
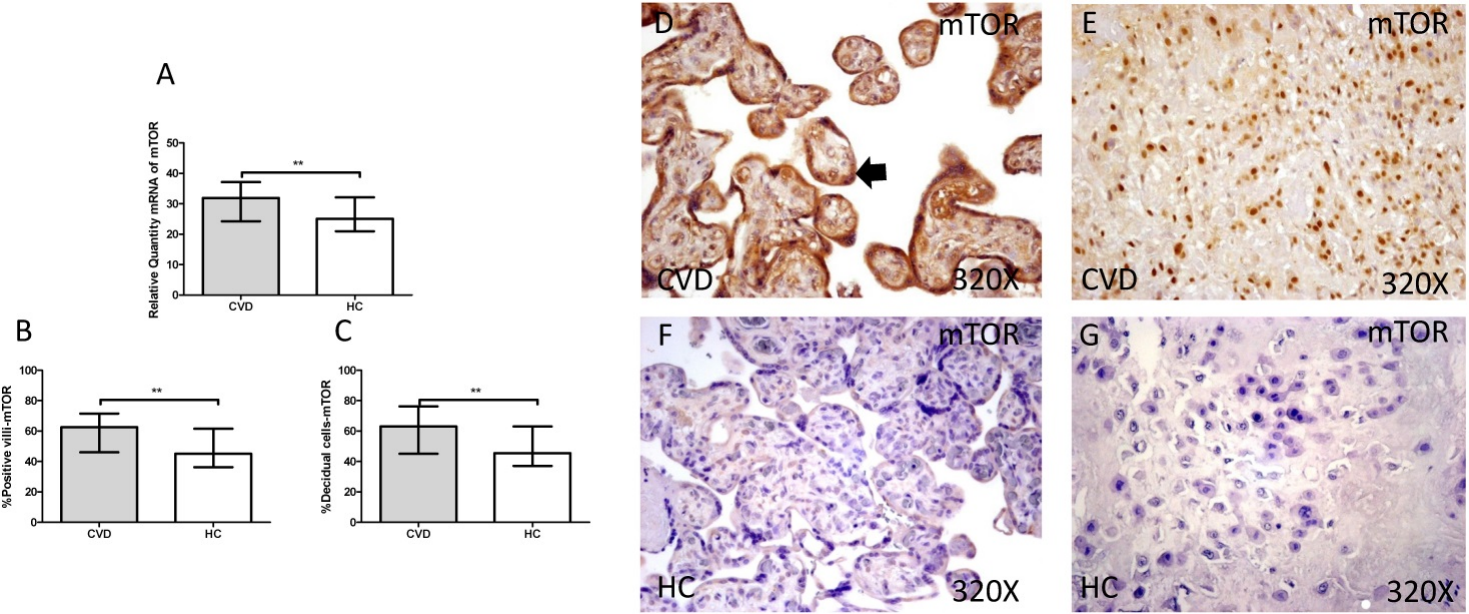
** A.** mRNA expression levels for Cyclin D1 by RT-qPCR.** B.** Percentage of placental villi with positive protein expression for Cyclin D1 by using immunochemistry techniques. **D-G.** Images where immunoexpression of Cyclin D1 is showed in placental villi (D and F) and in decidual cells (E and G). CVD=Women with diagnosed gestational chronic venous disease. HC= Venous control p<0.05 (*), p<0.01 (**), and p<0.001 (***). Arrow=shows positive expression of the protein.

Cyclin D1 showed a significant increase in its gene expression in placentas of women with gestational CVD in comparison to placentas of HC women (CVD=32.390 [29.145-49.170], HC=30.375 [5.812-38.770], *p=0.0028, Figure 11.A). The immunochemistry studies showed how Cyclin D1 expression was significantly greater in terms of expression percentage in placental villi of women with diagnosed gestational CVD in comparison to HC group (CVD=54.000 [28.000-91.000], HC=45.000 [10.000-77.000], **p=0.0010, Figure 11.B and D-F). In parallel, the increase in the percentage of Cyclin D1 protein expression was observed in decidual cells from placentas of women with CVD (CVD=77.000 [25.000-97.000], HC=34.500 [7.000-63.000], ***p<0.0001, Figure 11.C and E-G).

### 3.5. High expression of IL-4/IL-6 in placenta of women with gestational CVD

**Figure 12:**
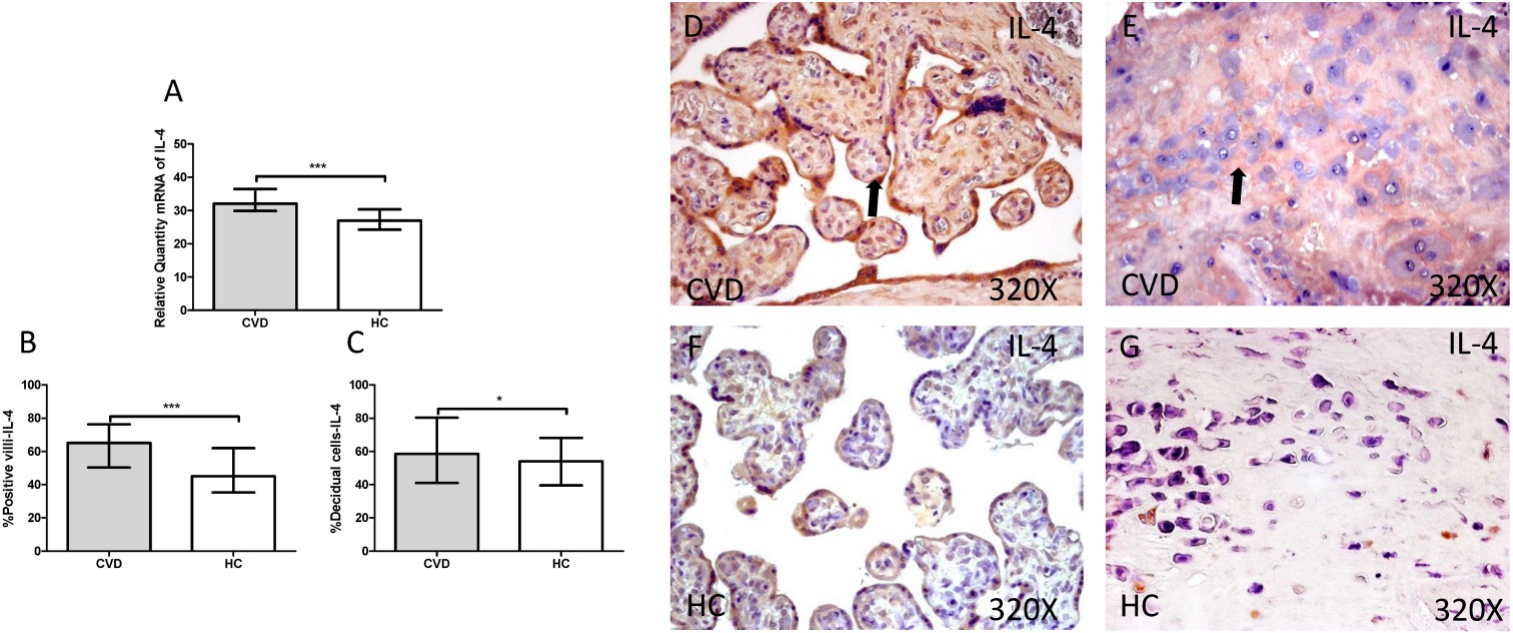
** A.** mRNA expression levels for IL-4 by RT-qPCR. B. Percentage of placental villi with positive protein expression for IL-4 by using immunochemistry techniques. D-G. Images where immunoexpression of IL-4 is showed in placental villi (D and F) and in decidual cells (E and G). CVD=Women with diagnosed gestational chronic venous disease. HC= Venous control p<0.05 (*), p<0.01 (**), and p<0.001 (***). Arrow=shows positive expression of the protein.

**Figure 13:**
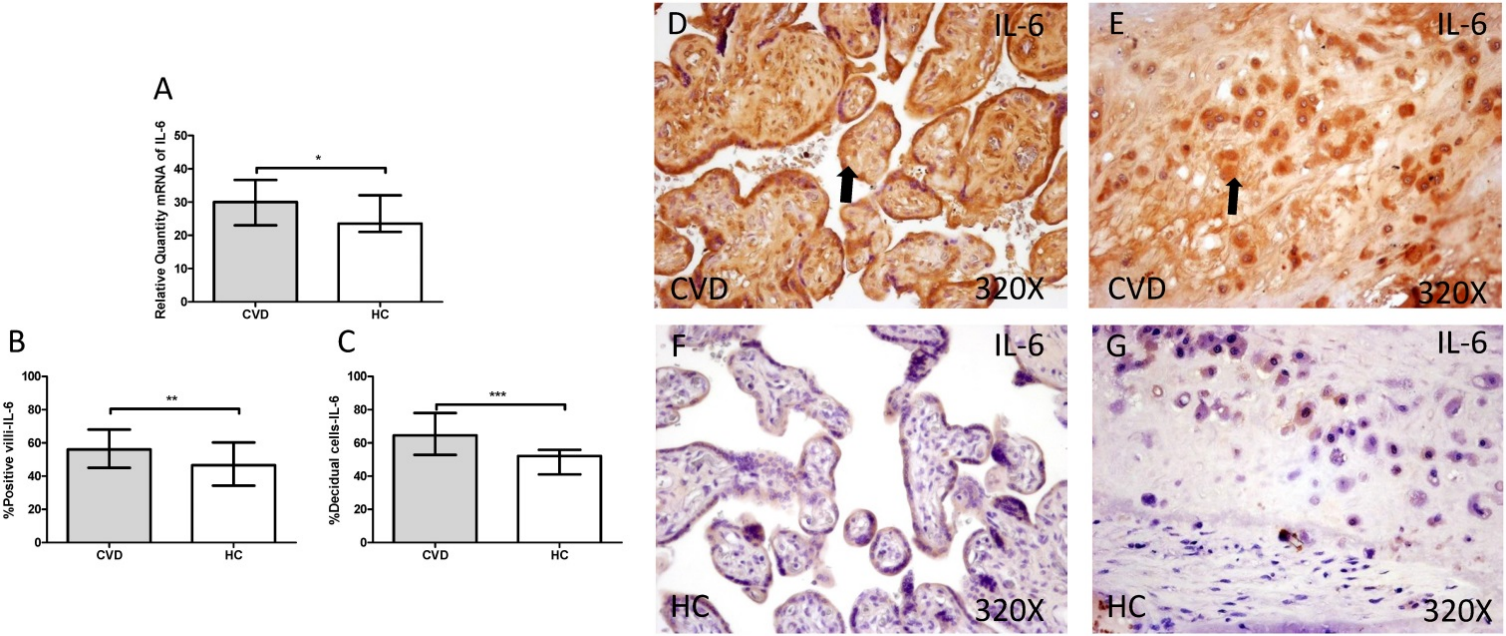
** A.** mRNA expression levels for IL-6 by RT-qPCR. **B.** Percentage of placental villi with positive protein expression for IL-6 by using immunochemistry techniques. **D-G.** Images where immunoexpression of IL-6 is showed in placental villi (D and F) and in decidual cells (E and G). CVD=Women with diagnosed gestational chronic venous disease. HC= Venous control p<0.05 (*), p<0.01 (**), and p<0.001 (***). Arrow=shows positive expression of the protein.

Gene expression studies using RT-qPCR showed an increase in IL-4 / IL-6 in CVD placentas [(IL-4 = CVD = 32,058 [21,987-49,369], HC = 26,974 [19,700-41,569], *** p <0.0001, Figure 12.A); (IL-6 = CVD = 30,016 [17,557-49,670], HC = 23,537 [12,489-43,894], * p = 0.0241, Figure 13.A)]. The study of protein expression in placental villi similarly showed an increase in IL-4 / IL-6 in women with gestational CVD [(IL-4 = CVD = 65,000 [21,000-99,000], HC = 45,000 [19,000-86,000], *** p <0.0001, Figure 12.B and D); (IL-6 = CVD = 56,000 [17,000-89,000], HC = 46,500 [19,000-81,000], ** p = 0.0031, Figure 13.B and D)]. This significant trend of increased protein expression was also shown in the decidual cells of placentas from women with gestational CVD [(IL-4 = CVD = 58,500 [29,000-97,000], HC = 54,000 [23,000-87,000], * p = 0.0483, Figure 12.C and E); (IL-6 = CVD = 64,500 [35,000-99,000], HC = 52,000 [12,000-98,000], *** p <0.0001, Figure 13.C and E)].

## 4. Discussion

In this paper, we have demonstrated that the placenta of CVD women show an abnormal villous cells behaviour with increased expression of the IGF-1/PAPP-A/STC-2 along with IRS-4, PI3K/Akt/mTOR activation pathways as well as the Wnt/ β-catenin canonical pathway accompanied by a proinflammatory environment.

Pregnancy is associated to development of CVD in a relevant group of previously healthy women. In the placenta of these women, there is evidence of cellular damage and tissue hypoxemic damage 13. However, the pathogenic mechanisms involved in alteration of the placenta villous cells of CVD remains undefined. It has been shown that the proliferation and differentiation of villous cells is regulated by different signals including IGF-1/PAPP-A/STC-2 pathway. IGF bioavailability is regulated in a paracrine manner in human placenta, controlling fetal and maternal tissue growth where it is bind to cells membrane glycosaminoglycans in placental villi, mainly in trophoblasts 41. Furthermore, in placenta as well as in other tissues, PAPP-A plays a key modulatory effect on IGF-1 activity. 42. PAPP-A provokes the lysis of IGFBP-4, releasing IGF-1 in the proximity of its receptor, promoting the cellular proliferation 43. This activation pathway is regulated by STC-2 that covalently binds to PAPP-A, inhibiting its mediated proteolysis of IGFBP-4. We have demonstrated in placenta villi and decidual cells of CVD an overexpression of PAPP-A and IGF with a reduction of that of STC-2. Interestingly, extracellular matrix (ECM) is a critical regulatory signal for PAPP-A abnormal expression and activation in certain pathological conditions including those of the placenta tissue 44, 45. Interestingly, a ECM remodelling has been observed in placenta of women with CVD 46. Thus, it is possible to suggest that in the placenta of women with CVD, ECM alteration might contribute to the induction of the observed PAPP-A overexpression. In addition, further mechanisms may play a pathogenic role in the induction of the IGF-1/PAPP-A/STC-2 pathway abnormalities found in placenta villi and decidual cells. Decrease in STC-2 has been related with ectopic calcifications in a broad range of tissues 47. Furthermore, the binding of calcium ions to the C-terminal domain PAPP-A play an important role in the interaction with IGFBP-4 and its proteolysis 48. An augmentation in placental villi calcifications has been described in CVD women 14. Thus, this excessive deposit of calcium in the placenta might also favour the proteolytic activity of PAPP-A. In addition, PAPP-A expression and activation is also regulated by the immunoinflammatory environment in damaged tissues. IL-6 appears to be a cytokine relevant for the IGF-1/PAPP-A/STC-2 regulation. 49 A direct association between PAPP-A levels and IL-6 has been described in fluid from inflammatory lesions, elucidating the link between inflammation and IGF-1 system. IL-4 also induce the expression of PAPP-A, acting synergistically with TGF-β 50. In this work, we have demonstrated an increased expression of IL-6 and IL-4 in villous placenta of CVD women supporting a potential additional mechanism for the observed increased IGF-1/PAPP-A activity. Interestingly, cytokine abnormal expression have been described in vascular damage of the placenta 51. Furthermore, PAPP-A has been associated to the induction of cytokines expression by the activation of IGF-1/PI3K pathways 25. Interestingly, it is possible to suggest that a pathogenic positive feedback between the cytokines IL6 and IL4 and IGF-1/PAPP-A pathway may be operating in the placenta villous.

IGF-1 plays a critical role in the activation of the PI3K/Akt/mTOR pathway. A key component in the IGF-1 signalling is the adaptor protein IRS-4. When IGF-1 binds to its receptor, IGF-1R, a tyrosine kinase receptor, phosphorylates itself and IRS-4, thus initiating the process of cell transduction 52. Afterwards, many researches have shown that IRS4 phosphorylates PI3K, therefore being a direct inductor of PI3K/Akt/mTOR pathway 53, 54. The overexpression of IGF-1 and PI3K/Akt/mTOR has been involved in the pathogenesis of vascular wall inflammation and aging 55, 56. Interestingly, In human placenta, enhanced IGF-1 levels have been associated to increased PI3K/Akt/mTOR pathway activity and subsequent alterations in placental protein synthesis, mitochondrial function and nutrient transport 57, 58. Our findings shown a marked overexpression of phosphorylated IRS-4 and PI3K/Akt/mTOR pathway in placenta villous of CVD women. These findings support that the observed increased levels of IGF1 induce an enhanced transduction signal of the IGF-1R receptor and of the PI3K/Akt/mTOR pathway in the villi cells. Thus, it is possible to suggest that this pathway activation may contribute to the cellular placenta damage and accelerated aging observed in CVD women. 59. In addition, the overexpression of hypoxia inducible factor 1- α (HIF-1α) found in placenta from CVD women may also be involved in the induction of this pathogenic PI3K/Akt/mTOR pathway in placenta villi. Moreover, PI3K/Akt/mTOR pathway is equally related with other pathological and vascular endothelial growing factor (VEGF) 60.

The development and normal functioning of placenta cells is complex and several regulatory pathways are involved. There evidence that in addition to PI3K/Akt/mTOR, Wnt/ β-catenin pathway plays a critical role in the regulation of villous palcenta cells 61. Pathological placenta conditions such as those observed in women with preeclampsia are associated to alteration in Wnt/ β-catenin pathway 33. Our results clearly show an increase in Wnt-1 and β-catenin expression in placenta villous cells of women with CVD. Wnt-1 is an important activator of canonical Wnt pathway that is physiologically highly expressed in trophoblasts in the early pregnancy, reducing its levels in the term, thus denoting the importance of this pathway in the initial stages of gestation 61. The overexpression of Wnt-1 may be explained by the stage of villous cellular damage observed in placenta of women with CVD (human pathology). It is possible to suggest that the observed Wnt-1 overexpression may support an involution of the placenta cells from CVD women. In addition, it has been well-established the interaction between IGF-1/PI3K signalling and canonical Wnt/ β-catenin pathway at different levels 62. After its activation in the cytoplasm, β-catenin translocates to the nucleus, forming a complex with other molecules and controlling the expression of a wide variety of genes such as c-myc and Cyclin D1, which will be essential in the cell fate determination 33. Our results show an important increase of both, Cyclin D1 and c-Myc in the placental tissue of women with CVD, in comparison with healthy subjects. The overexpression of both genes support the relevance of the described Wnt-1 and β-catenin expression in placenta villous cells from CVD women 63. It has been proposed a coordinated role of IGF-1/PI3K and β-catenin in promoting the expression of Cyclin D1 28. Interestingly, cyclin D1 promotes the regulation of placenta angiogenesis during the third trimester of gestation, a process increased in placenta of women with CVD 12, 64. c-Myc plays a critical role in the regulation of the proliferation and differentiation of trophoblast cells in physiological or pathophysiological conditions 65, 66. Taken together our results show that the villous cells of CVD women suffer an abnormal environment with increased signalization of the critical PI3K/Akt/mTOR and Wnt/ β-catenin pathways. The abnormal placenta tissue homeostasis is explained by an increases in IGF-1/PAPP-A/STC-2 stressor stimulation as well as in a local immune mediated proinflammatory ambiance. The described extracellular signalizations and intracellular abnormal regulatory events give lights for the understanding of the increased cellular damage and apoptosis and tissue remodelation observed in placenta from CVD women. An interesting point in future studies is to measure the systemic levels of these parameters to check their effects on the organism.

## 5. Conclusions

Our study is the first to describe that villous placenta cells of women presenting CVD show an alteration in the genic and protein expression of IGF-1/PAPP-A/STC-2 axis as well as its downstream signalling components such as IRS-4 PI3K/Akt/mTOR and of the Wnt/β-catenin pathway, with an increase in c-myc, Cyclin D. Altered villous environment is also observed with higher expression of PAPP-A and lower levels of its main inhibitor, STC-2, with increase in local IGF-1 bioavailability. Furthermore, Wnt expression is also increased and immunodisturbance with IL-4 and IL-6 levels is also found in placenta of CVD women. It remains elusive if this abnormalities are cause or consequence of the damage that suffer the placenta of women with CVD. Likewise, these alterations may have important implications in this organ, such as cell cycle dysregulation, or a homeostatic loss in the proliferative/apoptotic properties of the cells (graphical abstract). These changes could be associated with a premature aging and injuries in the tissue, hence being important to consider CVD as a possible damage for the placenta.

## Figures and Tables

**Table 1:** Primers utilized in RT-qPCR and temperature (Tm).

Gene	Seq. Fwd (5´→3´)	Seq. Rev (5´→3´)	Tm
**TBP**	TGCACAGGAGCCAAGAGTGAA	CACATCACAGCTCCCCACCA	60^O^C
**IGF-1**	GCTCTTCAGTTCGTGTGTGG	CGCAATACATCTCCAGCCTC	69^ O^C
**STC-2**	GCTCTCGGTCCCGTCAC	GACTCAGGAGAGCTCGACAC	51^ O^C
**PAPP-A**	CCCAGGCAGTCAGATCATCTTC	AGCTGCCCCTCAGCTTGA	52 °C
**Wnt-1**	CGATGGTGGGGTATTGTGAAC	CCGGATTTTGGCGTATCAGAC	60 °C
**β-catenin**	TTCGCCTTCACTATGGACTACC	GCACGAACAAGCAACTGAACTA	59 °C
**Cyclin D1**	CAGAAGTGCGAAGCTTAGGTCT	GTAGCAGGAGTAGTCCAGCGG	60 °C
**c-myc**	GGCTCCTGGCAAAAGGTCA	CTGCGTAGTTGTGCTGATGT	57 °C
**IRS-4**	CCCACACATGACCAGAGAGA	CTGACTGTCTGGGTTCAGCA	61 °C
**PI3K**	CTTGCCTCCATTCACCACCTCT	GCCTCTAATCTTCTCCCTCTCCTTC	60 °C
**Akt**	TGT CTC GTG AGC GCG TGT TTT	CCG TTA TCT TGA TGT GCC CGT C	60 °C
**mTOR**	ATCCAGACCCTGACCCAAAC	TCCACCCACTTCCTCATCTC	60 °C
**IL-4**	ACAGCCTCACAGAGCAGAAGACT	TGTTCTTGGAGGCAGCAAAGA	85 ºC
**IL-6**	AGTAGTGAGGAACAAGCCAGAG	TGGCATTTGTGGTTGGGTCA	60 °C

**Table 2:** Primary antibodies (A) and secondary (B) employed in immunochemistry studies, showing dilutions used and protocol specifications.

A	Antigen	Species	Dilution	Dealer	Protocol specifications
	**IGF-1**	Rabbit	1:100	Abcam (ab263903)	Sodium citrate 10 mM pH = 6 before incubation with blocking solution.
	**PAPP-A**	Mouse	1: 500	Abcam (ab52030)	Triton 0,1% in PBS, 10 minutes, before incubation with blocking solution.
	**STC-2**	Rabbit	1:300	Abcam (ab261915)	--------------------
	**Wnt-1**	Rabbit	1:50	Abcam (ab63934)	Triton 0,1% in PBS, 10 minutes, before incubation with blocking solution.
	**β-catenin**	Mouse	1:100	Abcam (ab231305)	--------------------
	**Cyclin D1**	Rabbit	1:300	Abcam (ab5262)	--------------------
	**c-myc**	Rabbit	1:250	Abcam (ab32072)	--------------------
	**IRS-4**	Rabbit	1:50	Abcam (ab5262)	Triton 0,1% in PBS, 10 minutes, before incubation with blocking solution.
	**PI3K**	Mouse	1:500	Abcam (ab86714)	--------------------
	**Akt**	Rabbit	1:1000	Abcam (ab8805)	--------------------
	**mTOR**	Rabbit	1:500	Abcam (ab1093)	--------------------
	**IL-4**	Mouse	1:250	Abcam (ab239508)	Triton 0,1% in PBS, 10 minutes, before incubation with blocking solution.
	**IL-6**	Mouse	1:200	Abcam, (ab9324)	Sodium citrate 10 mM pH = 6 before incubation with blocking solution.
**B**	**Antigen**	**Species**	**Dilution**	**Dealer**	**Protocol specifications**
	**IgG (Mouse)**	Goat	1:300	Sigma (F2012/045K6072 )	--------------------
	**IgG (Rabbit)**	Mouse	1:1000	Sigma (RG-96/ B5283)	--------------------
